# Chronic BPAF exposure differentially enhances fat deposition in mice fed normal or high-fat diets via lipid metabolism dysregulation

**DOI:** 10.3389/fendo.2025.1571076

**Published:** 2025-05-15

**Authors:** Ziquan Lv, Xinyue Xu, Zhi Tang, Yin Liang, Changfeng Peng, Yuxuan Wu, Dan Sang, Guixuan Jia, Xiaoxiao Hu, Ying Chen, Guangnan Liu, Dan Wang, Suli Huang, Yajie Guo

**Affiliations:** ^1^ School of Public Health, University of South China, Hengyang, China; ^2^ Division of Conservation and Application of Biological Resources, Shenzhen Center for Disease Control and Prevention, Shenzhen, China; ^3^ Department of Environmental and Occupational Health, School of Public Health, Guangdong Medical University, Dongguan, China; ^4^ Department of Emergency, The Eighth Affiliated Hospital, Sun Yat-Sen University, Shenzhen, China; ^5^ Department of Endocrinology, The Eighth Affiliated Hospital, Sun Yat-Sen University, Shenzhen, China; ^6^ School of Public Health, Shenzhen University Medical School, Shenzhen University, Shenzhen, China

**Keywords:** BPAF, high-fat diet, fat accumulation, lipid metabolism, obesity

## Abstract

**Background:**

Bisphenol AF (BPAF), an alternative to Bisphenol A (BPA), is increasingly utilized in various industrial applications, yet its toxicological profile remains incompletely understood. This study aims to investigate the impact of BPAF exposure on obesity and lipid metabolism in male mice subjected to either a normal chow diet (ND) or a high-fat diet (HFD).

**Methods:**

Mice were exposed to BPAF at a concentration of 100 μg/kg every other day for five months under different dietary conditions, and body weight, rectal temperature, and food intake were monitored regularly. After the mice were sacrificed, the hepatic lipid metabolism was analyzed by measuring serum, hepatic lipids and performing hepatic metabolomics; energy metabolism was elucidated by assessing thermogenic pathways in brown adipose tissue (BAT) and factors affecting ingestion in the hypothalamus; the development and pathways of obesity were indicated by exploring lipogenesis and lipolysis pathways and fat accumulation in white adipose tissue (WAT).

**Results:**

Histomorphometric analyses indicated that BPAF exposure induced drived fat deposition in white adipose tissue through adipocyte hypertrophy-mediated pathways in eWAT of ND and HFD mice, accompanied by weight gain in HFD mice. Energy metabolism analysis showed that BPAF exposure decreased resting body temperature and reduced thermogenic factor expression in BAT of ND and HFD mice, which may affect energy expenditure. Hepatic metabolomics analysis suggested that BPAF exposure interfered with hepatic lipid metabolism in ND and HFD mice, with elevated levels of hepatic triglycerides, total cholesterol, and free fatty acids in HFD mice. Transcript analysis revealed altered expression levels of genes regulating lipid metabolism in white adipose tissue of ND and HFD mice, with a down-regulation observed in p-HSL protein expression, indicative of a potential inhibition effects of BPAF on lipolysis signaling pathway.

**Conclusion:**

Chronic BPAF exposure differentially exacerbates fat deposition in mice fed normal or high-fat diets via affecting lipid metabolism. Given the widespread prevalence of obesity and the pervasive environmental presence of BPAF, our findings provide valuable insights into the metabolic toxicity of BPAF, thereby raise further concern on the safe utilization and precision prevention of this unique chemical.

## 1 Introduction

Obesity has become a serious public health problem in the present world, given its rising morbidity, excess mortality, and enormous medical and economic burden (1, 2). It is extensively linked to various chronic diseases, including, but not limited to, diabetes, fatty liver, hyperlipidemia, atherosclerosis, stroke and cancer. In particular, obesity in children and adolescents has evolved into an escalating epidemic, not only interfering with normal growth and metabolism but also persisting into adulthood, with profound long-term health consequences (3). The pathophysiological process of obesity is influenced by genetic, endocrine, behavioral, psychological, and environmental factors, and it is direct result of imbalance between energy intake and expenditure. Among these factors, diets high in fat and sedentary lifestyles are the most widespread causes of obesity (4). However, in recent years, the role of environmental chemicals in promoting metabolic disorders, including obesity, has also received increasingly more attention (5).

Bisphenol A (BPA) is a globally ubiquitous environmental chemical widely used in the production of plastics and resins, and has been detected in a variety of environmental matrices such as water, air, and soil, in human biological samples such as breast milk, plasma, and in other wild animals (6–9). A plethora of deleterious health effects have been demonstrated for BPA, including metabolic disturbance, endocrine disruption, hepatotoxicity, reproductive toxicity and neurotoxicity (10–13). Notably, exposure to BPA during the early stages of life (gestation and/or lactation) can interfere with the normal programming of the body’s systems (14). The epigenetic changes induced by this developmental exposure cause a significant increase in the risk of developing chronic diseases such as abnormalities of glucose and lipid metabolism and cardiovascular disease in adulthood (15, 16). These detrimental effects have prompted the search for safer alternatives. As a result, alternatives such as bisphenol AF (BPAF), bisphenol F (BPF), and bisphenol S (BPS) have been produced and put into use in large quantities. However, these analogs appear to present analogical or even more serious environmental threats and health risks than BPA (17–22).

Most bisphenols (BPs) have the potential to act as obesogens, a property that can be attributed to their capacity to interfere with the equilibrium of the endocrine system and energy metabolism as environmental hormonal pollutants (23). Epidemiologic studies have shown significant associations between BPs exposure and obesity (24–26). For example, a cross-sectional study showed a positive correlation between urinary levels of BPAF and indicators of childhood obesity, particularly in boys (27). Animal studies have shown that BPA and its substitutes (e.g., BPAF and BPF) interfere with hepatic lipid metabolism in male mice fed a high-fat diet, highlighting their potential role in inducing obesity (28). Moreover, perinatal exposure to these chemicals induces disorders of glucose and lipid metabolism, increased body weight and eWAT weight, and hepatic steatosis in offspring during adulthood (29–32). It has also been shown that BPS affects hypothalamic neuropeptides and interferes with feeding behavior, which might induce energy imbalance finally leading to obesity (33).

Although previous studies have revealed a strong association between BPs and obesity, studies on the obesogenic effects of long-term exposure to BPAF are still limited. The aim of this study was to investigate the role of long-term BPs (especially BPAF) exposure in the pathophysiological process of obesity under normal and high-fat dietary conditions. We wished to indicate whether obesity occurs by monitoring body weight changes, blood lipids, and white adipose tissue (WAT) weight (ratio); to explore lipid metabolism disorders by analyzing lipid synthesis and catabolism in epididymal white adipose tissue (eWAT) and subcutaneous white adipose tissue (sWAT) combined with hepatic lipid metabolism profiles; and to elucidate the imbalance of energy homeostasis by investigating thermogenic pathways in brown adipose tissue (BAT), food intake regulatory pathways in the hypothalamus, in conjunction with total energy intake of food and changes in resting body temperature. Our findings are expected to shed light on the health risks associated with BPAF exposure, especially its potential effects on obesity.

## 2 Materials and methods

### 2.1 Mice

Male C57BL/6J mice aged 8 weeks were brought from Guangdong Medical Laboratory Animal Center (Guangdong, China). All mice experiments were performed according to the guidelines of the Institutional Animal Care and Use Committee of Shenzhen Center for Disease Control and Prevention (Shenzhen CDC A2018019). Mice were fed either a normal chow diet (ND, 10 kcal% fat, Research Diets, D12450) or a high-fat diet (HFD, 60 kcal% fat, Research Diets, D12492) under controlled conditions of 22-25°C and a 12h light/dark cycle. Mice were provided with ad libitum access to food and water unless specified otherwise for experimental procedures.

### 2.2 Experimental design and treatment

After acclimatisation feeding, the mice were randomly divided into 8 groups of 10 mice each. Four of the groups (shown as ND-CON, ND-BPAF, ND-BPS, ND-BPF) were given a normal diet, while BPAF, BPF, and BPS were dissolved in olive oil (Macklin, O815211), respectively, and were administered to the mice by oral gavage at a concentration of 100 μg/kg every other day for five months. BPAF, BPF, and BPS were brought form Sigma-Aldrich (≥99% HPLC). Control mice were administered olive oil by gavage only. The other four groups (shown as HFD-CON, HFD-BPAF, HFD-BPS, HFD-BPF) of mice were given a high-fat diet, and other treatments as above.

### 2.3 Histological analysis of adipose tissue

The eWAT samples from mice were fixed in 4% paraformaldehyde overnight, embedded in paraffin, and sectioned. Sections were then subjected to hematoxylin and eosin staining for histological examination.

### 2.4 DNA content measurement

DNA was extracted from eWAT and sWAT using a DNA extraction buffer, and DNA concentration was quantified by NanoDrop spectrophotometer using the method of Ying Cheng et al (34).

### 2.5 TG, TC, FFA measurement

Liver cells lipids were extracted using chloroform-methanol. Serum and liver TG and TC levels were measured using the TG and TC kit (SSUF-C, Shanghai, China). Serum and liver FFA levels were determined using the FFA kit (Wako Pure Chemical Industries, Osaka, Japan).

### 2.6 Liver metabolites quantification

Targeted quantitative metabolomics analysis of liver tissue was performed on the XporeMET platform (Metabolo - Profile, Shanghai, China). Liver metabolites were quantitated using the ultrahigh-performance liquid chromatography-tandem mass spectrometry (UPLC-MS/MS) system (ACQUITY UPLC-Xevo TQ-S, Waters Corp., Milford, MA, USA). The raw data was performed by QuanMET software (v2.0, Metabo-Profile, Shanghai, China) and metabolites were peak-integrated, calibrated and quantified.

### 2.7 Western blot

Total proteins were extracted from tissue lysates (eWAT, sWAT, BAT), separated by 10% SDS-PAGE gels, and transferred onto PVDF membranes for western blotting. Primary antibodies were as follows: anti-FAS, anti-phospho-HSL (Ser660), anti-HSL, anti-CPT1A (all obtained from Cell Signaling Technology, Beverly, MA, USA), anti-SCD1, anti-UCP1 (Santa Cruz Biotechnology Inc, CA, USA), anti-β-Actin (Sigma, MO, USA).

### 2.8 Total RNA extraction and qRT-PCR analysis

Total RNA was extracted from BAT, eWAT, sWAT, and hypothalamus using TRIzol reagent (Invitrogen, USA). Synthesized cDNA from total RNA was used PrimeScript™ RT reagent kit (TaKaRa, Beijing, China). qRT-PCR analysis was performed using PowerUp™ SYBR™ Green Master Mix (ABI, USA) and Prism 7500 system (ABI, USA). Gene expression levels were normalized to GAPDH mRNA. Primers were synthesized by Sangon Biotechnology (Shanghai, China) and are shown in **Supplementary Table S1**.

### 2.9 Statistics

All the data are expressed as means ± standard error (SE). A two-tailed Student’s t-test (two groups) or one-way analysis of variance followed by a Student-Newman-Keuls (SNK) test (multiple groups) was used to assess significant differences, when the data conformed to normal distribution with chi-square. Conversely, nonparametric tests were used. P < 0.05 means statistically significant.

## 3 Results

### 3.1 BPAF treated mice showed increased body weight under HFD condition

**Figure 1:**
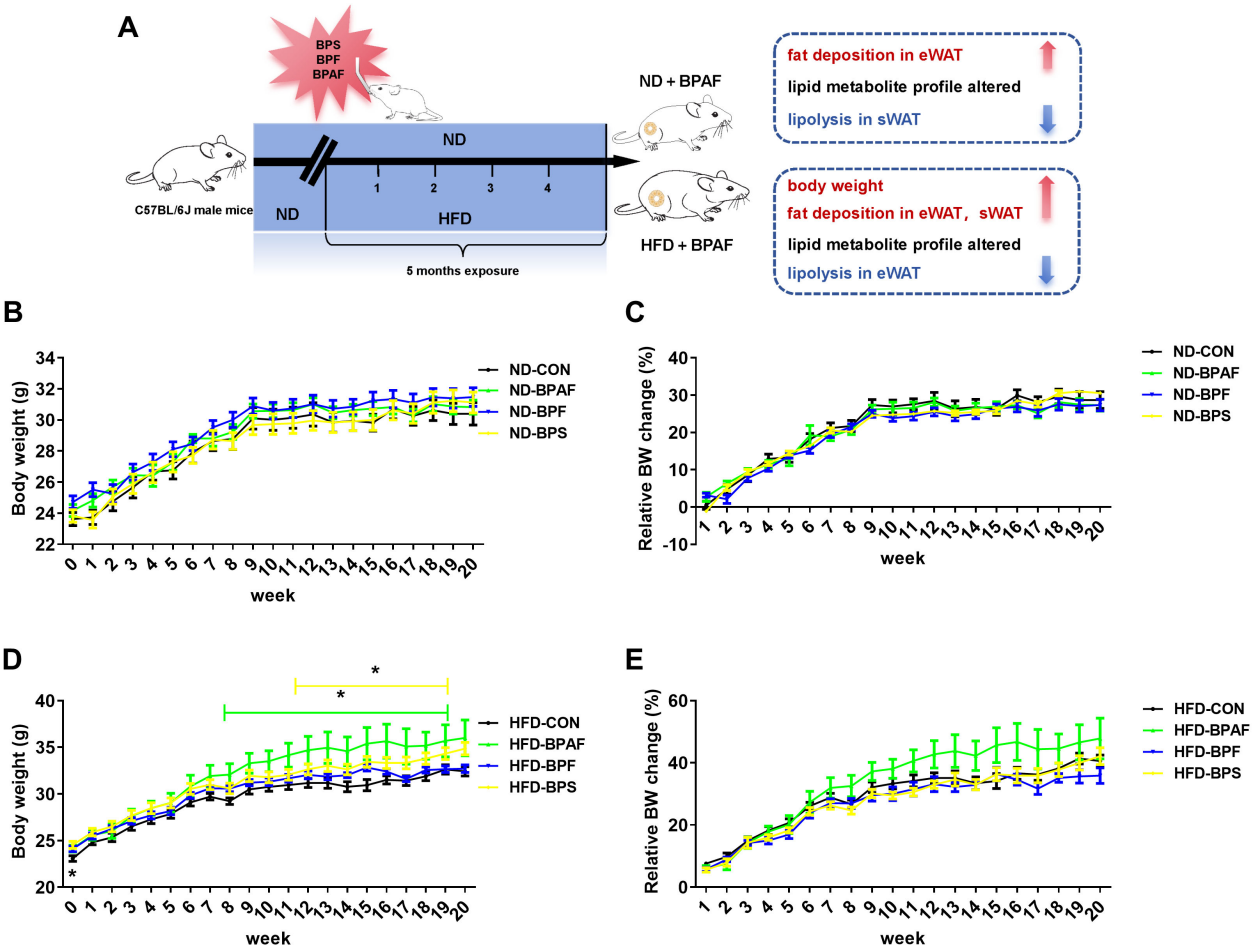
Effects of BPAF, BPF and BPS on body weight under ND and HFD conditions. C57BL/6j mice fed with ND or HFD were gavaged with 100 μg/kg of BPAF, BPF, or BPS for five months every other day, and body weights were measured weekly. **(A)** design of animal experiments in this study; **(B)** body weight of ND mice; **(C)** relative body weight change of ND mice; **(D)** body weight of HFD mice; **(E)** relative body weight change of HFD mice. N=8, data were expressed as means ± SE, one-way analysis of variance followed by Student-Newman-Keuls test, * means p <0.05.

BPA and its analogues have been implicated in adverse health effects and their association with obesity has been documented (35). To explore the effects of BPA analogues (BPAF, BPF, BPS) under varying nutritional conditions, male mice were subjected to 100 μg/kg of the chemicals by gavage every other day for 5 months, while feeding either a normal chow diet (ND) or a high-fat diet (HFD). The experimental setup is illustrated in **Figure 1A**. Results revealed that, under normal diet condition BPAF, BPF, and BPS treatments exhibited negligible effects on body weight (**Figure 1B**), with no significant differences observed in relative body weight changes (**Figure 1C**). However, under a high-fat diet, BPAF and BPS treatments, but not BPF, led to increased body weight (**Figure 1D**), although the relative body weight remained unchanged (**Figure 1E**).

### 3.2 BPAF induced fat accumulation in white adipose tissue

**Figure 2:**
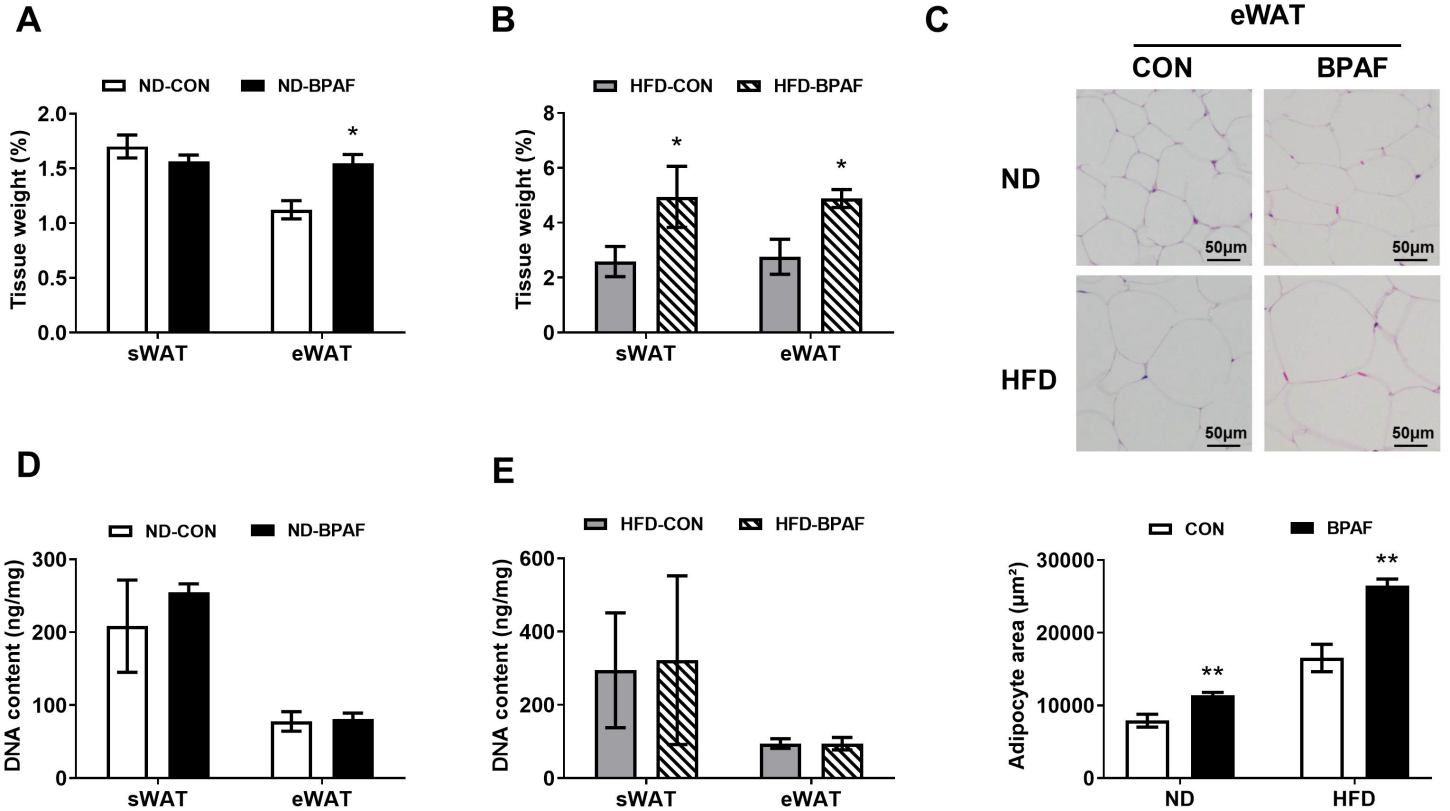
BPAF induced white adipose tissue fat accumulation. C57BL/6j mice fed with ND or HFD were gavaged with 100 μg/kg of BPAF for five months every other day, followed by examination of DNA content in eWAT and sWAT, weighing of eWAT and sWAT, detection of adipocyte size in eWAT. **(A)** sWAT and eWAT tissue weight (ratio) of ND mice treated with BPAF; **(B)** sWAT and eWAT tissue weight (ratio) of HFD mice treated with BPAF; **(C)** adipocyte size in eWAT of BPAF treated ND and HFD mice; *top*, representative images of H & E staining scale bar is 50 μm; *bottom*, area statistics of adipocyte; **(D)** DNA content per unit of tissue in sWAT and eWAT of BPAF treated ND mice; **(E)** DNA content per unit of tissue in sWAT and eWAT of BPAF treated HFD mice. N=6, data were expressed as means ± SE, two-tailed Student’s t test were used to analyze the difference between CON and BPAF group, * means p <0.05, ** means p <0.01.

Given the pronounced effects of BPAF on body weight gain, we further investigated its role in fat accumulation. BPAF treatment exhibited a significant increase in eWAT under normal chow diet condition (**Figure 2A**). Conversely, no discernible effect was observed on the weight of sWAT (**Figure 2A**). However, mice fed a high-fat diet displayed increased fat deposition in both eWAT and sWAT following BPAF exposure (**Figure 2B**). Consistently, histological analysis confirmed that adipocytes in eWAT were larger in the BPAF-treated group of mice than in the control mice, both under normal and high-fat diet conditions (**Figure 2C**). Additionally, DNA content in sWAT and eWAT exhibited no significant differences in mice subjected to either normal diet or high-fat diet with BPAF treatment (**Figures 2D, E**). This suggests that BPAF exposure did not cause an increase in the number of adipocytes in WAT of mice. Collectively, our mechanistic analyses establish that BPAF exposure drived fat deposition in eWAT through adipocyte hypertrophy-mediated pathways, rather than hyperplastic expansion.

### 3.3 Impact of BPAF exposure on energy homeostasis

**Figure 3:**
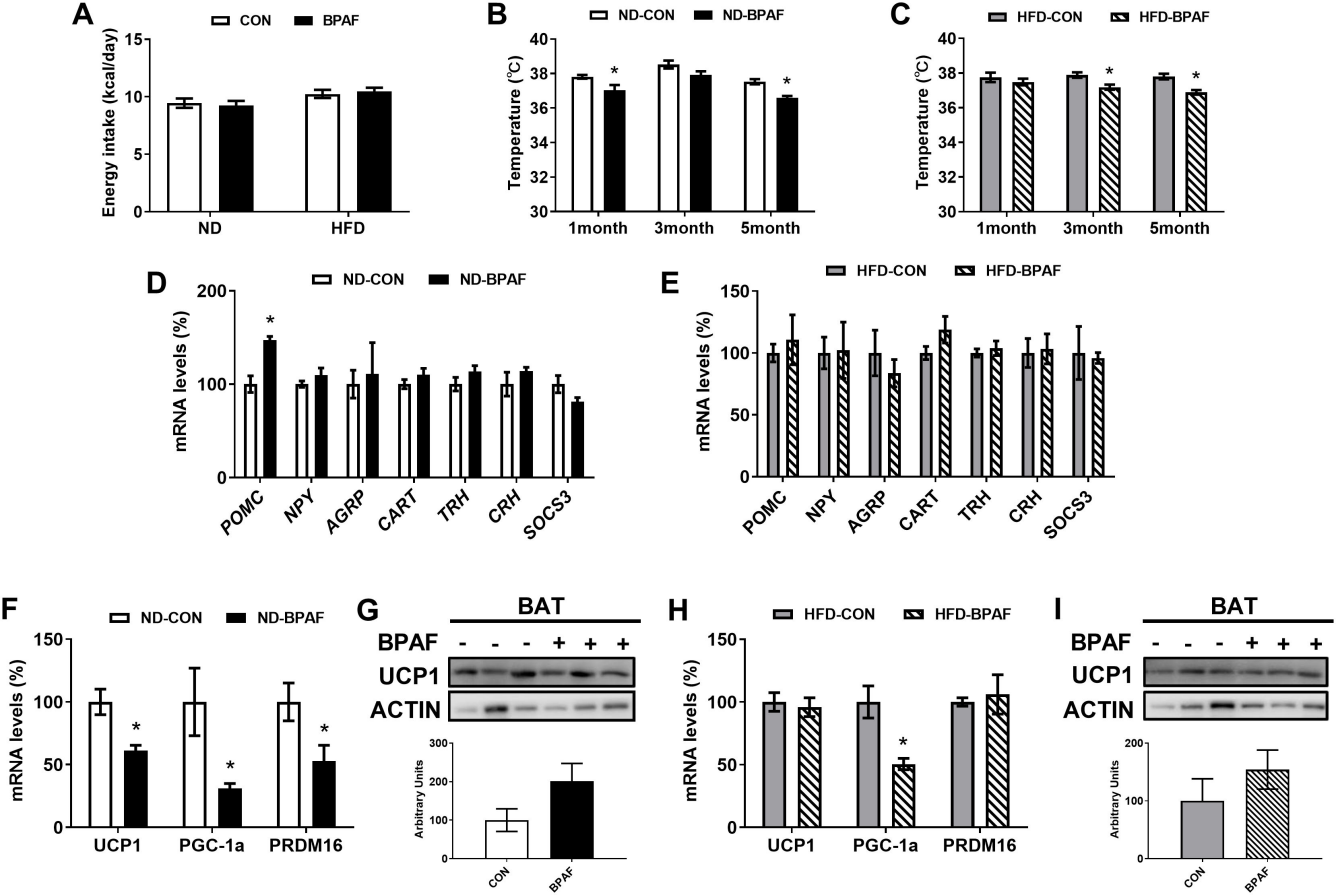
Impact of BPAF exposure on energy homeostasis. C57BL/6j mice fed with ND or HFD were gavaged with 100 μg/kg of BPAF for five months every other day, and monitored for body temperature and energy intake, followed by examination of gene and protein expression of relevant factors in BAT and hypothalamus. **(A)** changes in daily energy intake in ND and HFD mice; **(B)** rectal temperature of ND mice at different times of the test; **(C)** rectal temperature of HFD mice at different times of the test; **(D)** POMC, NPY, AGRP, CART, TRH, CRH, SOCS3 gene expression in hypothalamus of ND mice; **(E)** POMC, NPY, AGRP, CART, TRH, CRH, SOCS3 gene expression in hypothalamus of HFD mice; **(F)** UCP1, PGC1a, PRDM16 gene expression in BAT of ND mice; **(G)** UCP1 protein expression in BAT of ND mice; **(H)** UCP1, PGC1a, PRDM16 gene expression in BAT of HFD mice; **(I)** UCP1 protein expression in BAT of HFD mice. *top*, western blots; *bottom*, quantitative measurements of UCP1 protein relative to β-actin protein. N=6, data were expressed as means ± SE, two-tailed Student’s t test were used to analyze the difference between CON and BPAF group, * means p <0.05.

Imbalances in energy homeostasis play a pivotal role in fat accumulation (4). To evaluate the effects of BPAF on energy intake, we treated different nutritional mice with BPAF, and results showed that BPAF exposure had no influence on the total energy intake from food (**Figure 3A**). Rectal temperatures in the resting state were significantly lower in the ND-BPAF group of mice at one month and five months from the start of the experiment compared to the ND-CON group of mice (**Figure 3B**). Compared with HFD-CON mice, rectal temperatures in the resting state of mice were measured to be lower at 3 and 5 months after BPAF exposure (**Figure 3C**). The hypothalamus, as a neuroendocrine regulatory center, plays a crucial role in the development of obesity (36). We evaluated the expression levels of key factors regulating feeding behavior and energy metabolism in hypothalamus, including proopiomelanocortin (POMC), neuropeptide (NPY), agouti-related protein (AGRP), cocaine and amphetamine-regulated transcript (CART), thyrotropin releasing hormone (TRH), corticotropin releasing hormone (CRH), and suppressor of cytokine signaling 3 (SOCS3) (36). Under normal diet condition, POMC expression was significantly increased (**Figure 3D**), while no changes were observed in HFD mice (**Figure 3E**) after BPAF treatment. The thermogenic function of BAT is not only involved in body temperature maintenance, but also influences obesity by regulating energy distribution balance (37, 38). We found that BPAF exposure decreased the expression levels of genes associated with thermoregulation, including uncoupling protein 1 (UCP1), peroxisome proliferator-activated receptor gamma co-activator-1 alpha (PGC1α) and PR domain containing 16 (PRDM16) (39) in BAT of mice subjected to normal diet (**Figure 3F**), although UCP1 protein expression was not changed (**Figure 3G**). Only PGC1α gene expression exhibited a significant decrease, while UCP1 and PRDM16 showed no significant differences in HFD mice after BPAF treatment (**Figures 3H, I**).

### 3.4 Impact of BPAF exposure on lipid metabolites

**Figure 4:**
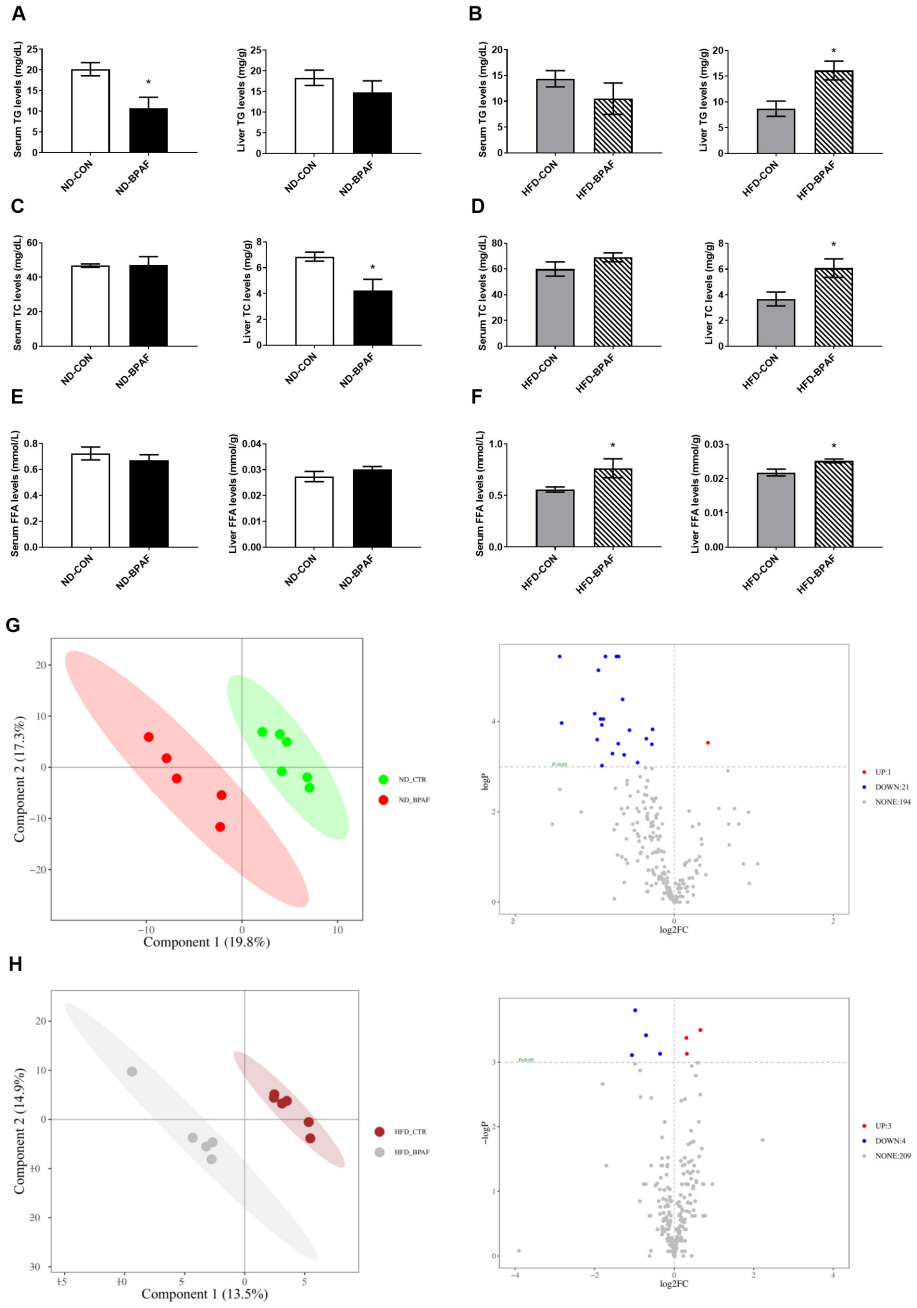
Impact of BPAF exposure on lipid metabolites. C57BL/6j mice fed with ND or HFD were gavaged with 100 μg/kg of BPAF for five months every other day, followed by examination of liver and serum TG, TC, FFA levels and analysis of liver metabolomics. **(A)** serum and liver TG levels in ND mice; **(B)** serum and liver TG levels in HFD mice; **(C)** serum and liver TC levels in ND mice; **(D)** serum and liver TC levels in HFD mice; **(E)** serum and liver FFA levels in ND mice; **(F)** serum and liver FFA levels in HFD mice; **(G)** analysis of liver metabolomics in ND mice; **(H)** analysis of liver metabolomics in HFD mice. left, PLS-DA score plots; right, volcano plots. N=6, data were expressed as means ± SE, two-tailed Student’s t test were used to analyze the difference between CON and BPAF group, * means p <0.05.

Disorders of lipid metabolism are commonly associated with obesity in a subset of obese patients. To elucidate the role of BPAF in lipid metabolism regulation, we first analyzed liver and serum lipids levels in mice. Under normal diet condition, serum TG levels were decreased while liver TG levels remained unchanged (**Figure 4A**). Serum TC levels showed no differences, whereas liver TC levels were decreased in BPAF-treated mice (**Figure 4C**). Serum and liver FFA levels exhibited no significant changes (**Figure 4E**). Conversely, under high-fat diet condition, BPAF-treated mice displayed elevated liver TG, TC, and FFA levels, as well as increased serum FFA levels, with no differences observed in serum TG and TC levels compared to BPAF untreated mice (**Figures 4B, D, F**). Additionally, we performed metabolomic analysis of liver tissue, Partial Least Squares Discriminant Analysis (PLS-DA) score plots showed clear separation of control and BPAF, both in ND and HFD mice (**Figures 4G, H**), suggesting that BPAF exposure disrupted hepatic metabolic profiles. Alterations in liver lipid metabolites were also quantified, with a distinct profile of unregulated and downregulated metabolites in both normal diet and high-fat diet mice following BPAF exposure. In ND mice, there were 1 metabolite up-regulated and 13 metabolites down-regulated (**Figure 4G**) and in HFD mice, there were 3 metabolites up-regulated and 4 metabolites down-regulated (**Figure 4H**). Detailed of differential lipid metabolites were presented in **Tables 1**, **2**. These changed molecules indicated the regulated role of BPAF on lipid metabolism homeostasis.

**Table 1:** Differential hepatic lipid metabolites (μM/g).

Metabolites	ND-CON	ND-BPAF	Change
Myristoleic acid	0.0085 ± 0.0008	0.0114 ± 0.0020	up
L-Alpha-Aminobutyric acid	0.0106 ± 0.0083	0.0030 ± 0.0009	down
Citraconic acid	0.0012 ± 0.0005	0.0006 ± 0.0001	down
Adipoylcarnitine	0.0035 ± 0.0011	0.0011 ± 0.0002	down
2-Hydroxybutyric acid	0.1463 ± 0.0399	0.0756 ± 0.0216	down
Fumaric acid	0.0075 ± 0.0017	0.0048 ± 0.0012	down
Octanoylcarnitine	2.17E-05 ± 7.53E-06	8.2E-06 ± 4.02E-06	down
DPAn-6	1.0266 ± 0.3209	0.5777 ± 0.0814	down
2-Methylbutyroylcarnitine	0.0013 ± 0.0004	0.0006 ± 0.0002	down
Decanoylcarnitine	8.33E-05 ± 4.23E-05	3.00E-05 ± 8.37E-06	down
Glutarylcarnitine	0.1220 ± 0.0042	0.0065 ± 0.0019	down
L-Homocitrulline	0.0025 ± 0.0003	0.0021 ± 0.0001	down
2-Methyl-4-pentenoic acid	0.0011 ± 0.0002	0.0007 ± 0.0002	down
Maleic acid	0.0212 ± 0.0063	0.0123 ± 0.0040	down

**Table 2:** Differential hepatic lipid metabolites in HFD mice (μM/g).

Metabolites	HFD-CON	HFD-BPAF	Change
L-pipecolic acid	0.0070 ± 0.0014	0.0087 ± 0.0006	up
Azelaic acid	0.0009 ± 0.0001	0.0011 ± 0.0002	up
Rhamnose	0.0181 ± 0.0031	0.0215 ± 0.0033	up
DPAn-6	1.1505 ± 0.4334	0.5831 ± 0.1444	down
10,13-Nonadecadienoic acid	0.0289 ± 0.0067	0.0177 ± 0.0076	down
Glycylproline	0.0479 ± 0.0051	0.0374 ± 0.0082	down
Murocholic acid	0.0009 ± 0.0004	0.0004 ± 0.0003	down

### 3.5 Impact of BPAF exposure on lipid metabolism in WAT

**Figure 5:**
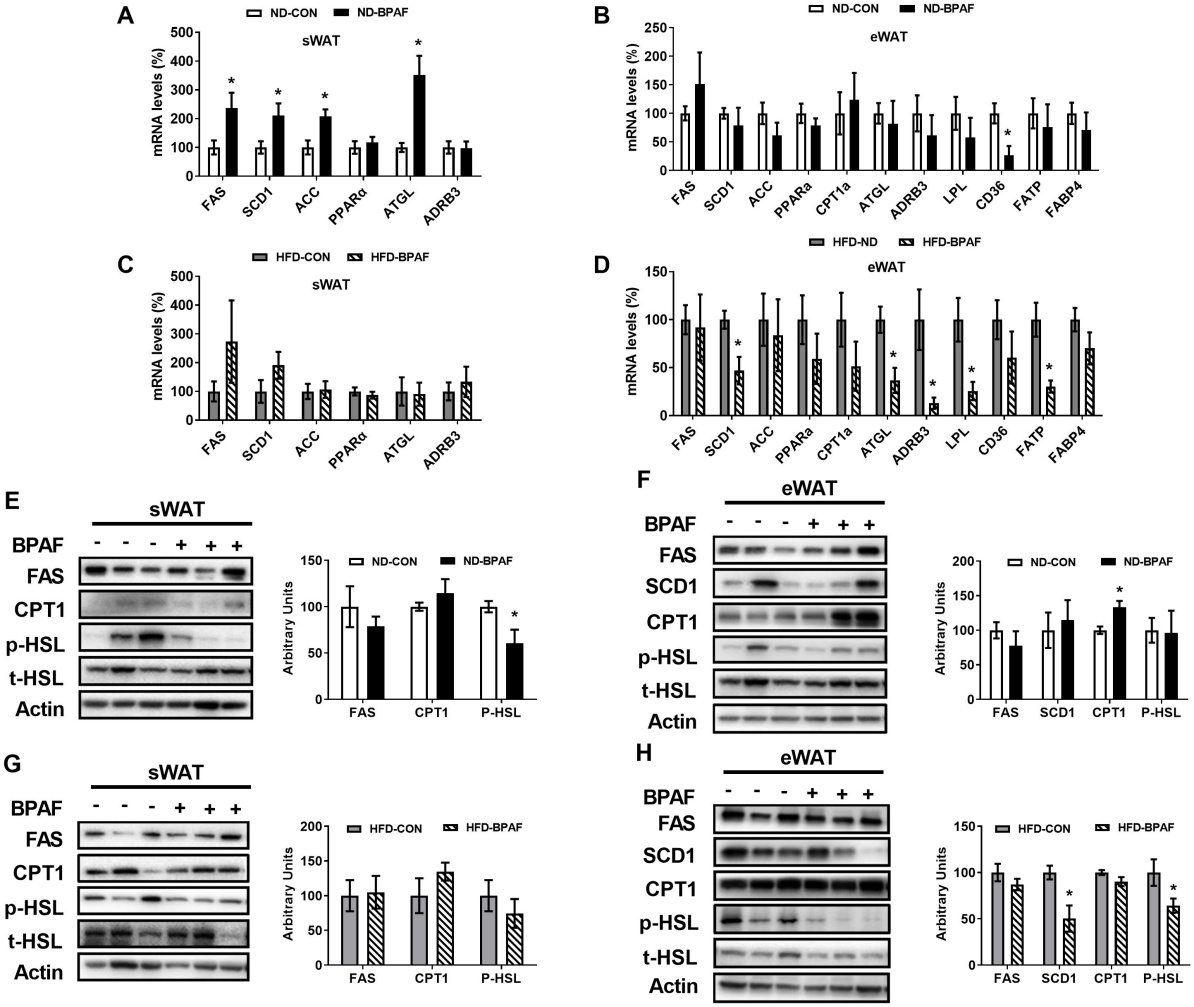
Impact of BPAF exposure on lipid metabolism in WAT. C57BL/6j mice fed with ND or HFD were gavaged with 100 μg/kg of BPAF for five months every other day, followed by examination of gene and protein expression of factors related to lipogenesis and lipolysis in eWAT and sWAT. **(A)** FAS, SCD1, ACC, PPARα, ATGL, ADRB3 gene expression in sWAT of ND mice; **(B)** FAS, SCD1, ACC, PPARα, ATGL, ADRB3, LPL, CD36, FATP, FABP4 gene expression in eWAT of ND mice; **(C)** FAS, SCD1, ACC, PPARα, ATGL, ADRB3 gene expression in sWAT of HFD mice; **(D)** FAS, SCD1, ACC, PPARα, ATGL, ADRB3, LPL, CD36, FATP, FABP4 gene expression in eWAT of HFD mice; **(E)** FAS, CPT1, p-HSL (Ser660) protein expression in sWAT of ND mice; **(F)** FAS, SCD1, CPT1, p-HSL (Ser660) protein expression in eWAT of ND mice; **(G)** FAS, CPT1, p-HSL(Ser660) protein expression in sWAT of HFD mice; **(H)** FAS, SCD1, CPT1, p-HSL (Ser660) protein expression in eWAT of HFD mice. *left*, western blots; *right*, quantitative measurements of FAS, SCD1, CPT1 protein relative to β-actin protein, quantitative measurements of p-HSL protein relative to t-HSL protein. N=6, data were expressed as means ± SE, two-tailed Student’s t test were used to analyze the difference between CON and BPAF group, * means p <0.05.

Furthermore, we assessed the expression levels of lipid metabolism-related factors in WAT. Under normal diet condition, genes associated with enzymes related to lipogenesis (40), including fatty acid synthase (FAS), stearoyl-Coenzyme A desaturase 1(SCD1) and acetyl-CoA carboxylase (ACC) were upregulated in sWAT following BPAF treatment (**Figure 5A**). Genes related to fatty acid oxidation and lipolysis (41, 42), peroxisome proliferator-activated receptor alpha (PPARα) and adrenergic receptor bata 3 (ADRB3) was not changed (**Figure 5A**). However, the gene of adipose triglyceride lipase (ATGL), which is involved in lipolysis, also exhibited increased expression in BPAF exposure mice (**Figure 5A**). In eWAT, only the gene expression of fatty acid uptake-associatedcluster of differentiation 36 molecule (CD36) was downregulated (**Figure 5B**). HFD mice displayed no changes in gene expressions in sWAT(**Figure 5C**), while BPAF treatment resulted in significant decreases in the expression of genes associated with lipid synthesis (SCD1), lipolysis (ATGL, ADRB3 and lipoprotein lipase (LPL)), and fatty acid transport (fatty acid transport protein (FATP)) in eWAT (**Figure 5D**). Protein expression analysis revealed reduced levels of phosphorylated hormone-sensitive lipase (p-HSL) in sWAT of ND mice following BPAF treatment (**Figure 5E**), while no changes were observed in HFDmice (**Figure 5G**). In eWAT, BPAF treatment led to increased CPT1 protein levels (**Figure 5F**), whereas decreased expression levels of SCD1 and p-HSL were observed in HFD mice (**Figure 5H**).

## 4 Discussion

In this study, we firstly examined the effects of BPs exposure on the weight change of male mice subjected to normal diet or high-fat diet. Our findings indicate that a relatively low dose of BPAF and BPS exposure, but not BPF, led to increased body weight in HFD mice, with BPAF exhibiting a more pronounced effect compared to BPS. However, no significant changes were observed in ND mice. Given that the relationship between BPF and BPS with body weight in mice has also been measured in our previous studies (43, 44). The effect of BPAF on obesity, however, remains unclear. Therefore, we discuss and showed only BPAF results in our study. BPAF exposure induced fat deposition in sWAT and eWAT of HFD mice and eWAT of ND mice, accompanied by an increase in adipocyte volume in eWAT. Altered core temperature in the resting state of ND and HFD mice, as well as decreased expression of thermogenic factors in the BAT. Notably, alterations in lipid metabolism were evident in both liver and WAT following BPAF exposure. Specifically, hepatic TG, TC, and FFA levels were elevated in HFD mice after BPAF treatment, accompanied by alterations in hepatic lipid metabolite profiles. Furthermore, BPAF induced changes in the expression of factors related to lipogenesis and hydrolysis that contribute to fat accumulation in eWAT and sWAT. These findings indicate that BPAF exposure disrupts metabolic functions of liver and adipose tissue and outline a possible mechanism that links BPAF to the prevalence of obesity and other metabolic disorders.Based on the dosage range selected in the previous studies and the legal dosage limits released by the government supervision department, we selected the concentration of BPAF at 100 µg/kg every other day in this study.This dosage is close to two equivalents of a temporary tolerable daily intake (t-TDI) of BPA (4 mg/kg bw per day) recommended by European Food Safety Authority in 2015 (7). In previous researches, the exposure dosage of BPAF applied in mice experiments ranged from 0.05mg/kg body weight/day to 5mg/kg body weight/day for days or weeks (28, 32, 45, 46). Some studies investigated the effects of BPAF on liver lipid metabolism and liver damage (28, 30, 32). Others explored the role of BPAF in immune system (22, 46), reproductive system (45) or neurotoxicity (45, 46). Although extensive studies have shown that BPAF increases the risk of obesity, the specific effects of sustained exposure to BPAF on the development of obesity and the mechanism of action remain to be clarified (20, 24, 27, 47). Our current study systematically reveals that chronic exposure to bisphenol AF (BPAF) disrupts lipid metabolism thereby leading to fat accumulation and somewhat reduces energy expenditure, with this effect being significantly amplified under high-fat diet conditions.

BPAF-induced obesity in mice might be influenced by multiple factors, such as the exposure pattern, dosage and duration, and dietary conditions. In our study, 100 µg/kg every other day of BPAF exposure for five months resulted in fat accumulation in both ND and HFD mice, with a significant increase in body weight in HFD mice. However, in a previous study, mice exposed to 50 μg/kg/day of BPAF for 8 weeks under high-fat conditions showed no significant change in body weight (28). In addition, two previous studies have shown that the sex of the subject and the window of exposure (perinatal, adolescent, adulthood) are also key determinants in modulating the hypertrophic effects of BPAF (30, 32). A study using mature human adipocytes also showed that exposure to bisphenol AF mediates inflammatory signaling pathways that disrupt adipocyte metabolism (48).

Energy balance, comprising energy intake and expenditure, plays a crucial role in the development of obesity (4). Previous studies have found that postweaning exposure to BPAF and other bisphenol analogs induced an increase in energy intake in subjects, but this phenomenon was not observed in the present study, which may be related to the specificity of BPAF as well as the different age of exposure (33, 49). Appetite and feeding behavior are controlled by metabolic signaling pathway as well as neuroendocrine system (36). The expression of neuropeptide in the hypothalamus were investigated, including the anorexigenic neuropeptides POMC and CART, the appetite-stimulating neuropeptides AgRP and NPY, two neuroendocrine hormones, TRH and CRH, as well as the leptin-associated factor SOCS3 (36). The results showed that BPAF induced elevated expression of the anorexigenic neuropeptide POMC in ND mice, but no significant changes in other factors. Another study also showed that BPAF upregulates the expression of POMC, which induced sWAT browning, a means of maintaining body temperature and regulating energy metabolism in mice (46). Consequently, it can be concluded that the neuroendocrine disrupting effects of low-dose BPAF exposure should not be disregarded. In addition, the thermogenic function of BAT is critical for energy expenditure and maintenance of resting body temperature (37, 38). However, core body temperature decreased in both ND and HFD mice after BPAF exposure. Meanwhile, the expression of UCP1, PGC-1α, and PRDM16 in the BAT, critical thermogenic tissue, was decreased, indicating that its thermogenic function was inhibited. BPAF may impair energy expenditure in mice to some extent, thereby contributing to the development of fat accumulation and obesity.

Lipids are important biomolecules that play key biophysical roles in material metabolism, energy balance, and physiological signal transduction (50).The effects of BPAF exposure on hepatic and serum lipid homeostasis appear to be inconsistent across dietary conditions. We found that BPAF exposure downregulated hepatic TC and serum TG levels in ND mice but upregulated hepatic TG, TC and FFA levels as well as serum FFA levels in HFD mice. Another study in which mice were treated with BPAF at 0.05 mg/kg bw/day for 8 weeks showed a decrease in hepatic TG and TC levels in HFD mice, but transcriptomics results showed up-regulation of differentiated expressed genes (DEGs) involved in lipogenesis and down-regulation of DEGs involved in the lipolytic pathway (28). This discrepancy might be largely ascribed to different exposure doses, durations and frequencies. However, there is no doubt that BPAF treatment interferes with hepatic lipid metabolism. *In vitro* experiments have also demonstrated that BPAF interferes with intracellular lipid homeostasis, with lower concentrations of BPAF maximizing intracellular lipid levels during adipocyte differentiation. In contrast, BPAF-glucuronide, the main metabolite of BPAF *in vivo*, has a different mode of action, increasing lipid accumulation in a dose-dependent manner (51, 52).

Dysregulation of hepatic lipid metabolism is a key factor in distinguishing between healthy and metabolically diseased obesity and suggests a mechanism by which BPAF induces obesity (4, 53). In this study, we examined hepatic metabolites using UPLC-MS/MS, and a total of more than 200 lipid metabolites were detected. The hepatic lipid metabolite profiles in ND and HFD mice were significantly changed after BPAF treatment. Almost all altered hepatic lipid metabolites were down-regulated in BPAF exposed ND mice, whereas altered hepatic lipid metabolites in HFD mice were either up-regulated or down-regulated. Docosapentaenoic acid (22n-6) (DPAn-6) was the lipid metabolite co-reduced in the livers of ND and HFD mice. DPAn-6 is able to increase the activity of lipolysis-related enzymes by inhibiting the activity and expression of lipase-producing enzymes in mouse livers, which in turn attenuated hepatic steatosis (54). At the same time, DPAn-6 has significant anti-inflammatory effects (55). The decrease of DPAn-6 might be a result of the abnormalities in lipid metabolism induced by BPAF.

In addition, we found that several differential hepatic lipid metabolites in BPAF exposed HFD mice were strongly associated with obesity pathogenesis, including L-pipecolic acid, Murocholic acid, Glycylproline and 10,13-Nonadecadienoic acid. In a previous study, serum etabolite pipecolic acid levels were significantly increased in obese mice (56), which is consistent with what we observed in HFD mice treated with BPAF but not in ND mice. This indicates that lipid metabolism disorders are more likely to be a synergistic effect induced by BPAF exposure in combination with high-fat diet consuming. Similarly, it has been shown that murocholic acid has a positive effect on ameliorating obesity and metabolic disorders (57), but we found that BPAF exposure led to a decrease in murocholic acid in HFD mice. A study in male adolescents showed that urinary glycylproline levels were negatively correlated with pubertal body mass index (BMI), and a decrease in this index is a sensitive biomarker of obesity (58). Furthermore, FP fermentation broth of C. leptum (FPF), which was rich in glyceroproline, was able to mediate the AMPK pathway to block hepatic fatty acid synthesis and accelerate the oxidative catabolism of fatty acids, thus effectively reducing body fat in obese mice (59). The above results suggest that simultaneous exposure to HFD and BPAF may aggravate the burden on hepatic lipid metabolism and more easily induce obesity.

WAT is a core storage site for fat (mainly TG) and its quality reflects fat deposition. This energy buffering capacity maintains systemic energy homeostasis by regulating lipid metabolism. Lipid synthesis, lipid hydrolysis and lipid oxidation are the basic processes of lipid metabolism (60). We examined the expression of factors related to lipid metabolism in different adipose tissues. BPAF induced up-regulation of the expression of genes related to fatty acid synthesis (Fas, Scd 1, and Acc) and decreased phosphorylation of HSL, a key enzyme that promotes lipid hydrolysis, in sWAT of ND mice. These results indicate that the risk of fat accumulation was dramatically increased, although the weight of sWAT in ND mice was not significantly elevated. In contrast, the expression of the lipolytic enzyme ATGL was upregulated, which might be an adaptive regulation to maintain lipid homeostasis. Furthermore, the diminished CD36 expression and augmented CPT1 expression in eWAT of ND mice imply that the uptake and transport of long-chain fatty acids is constrained, and that β-oxidation is augmented, consequently leading to a reduction in lipid synthesis and accumulation. Other studies have also observed that perinatal BPAF exposure resulted in up-regulation of CPT1a expression in the adult liver of offspring (30). Concomitantly, in conjunction with the elevated weight of eWAT and the increased adipocyte volume, we conclude that these changes are negative feedback regulated, reducing lipogenesis and accelerating lipolysis. The above suggest that some protective mechanisms exist in ND mice to cope with BPAF-induced lipid metabolism disorders.

In contrast, BPAF elicited a decrease in the expression of ATGL, ADRB3, LPL, and FATP, along with a reduction in HSL phosphorylation (p-HSL) in eWAT of HFD mice, suggesting inhibition of fatty acid catabolism, transport, and utilization. BPAF interfered with lipid depletion processes. Whereas the decreased gene and protein expression of SCD1 was presumably related to a compensatory mechanism that reduces further lipid synthesis. However, the elevated eWAT weight indicated this compensation was not sufficient to completely prevent the occurrence of obesity.

This study has several limitations. First, we did not monitor basal metabolic rate (BMR) by indirect calorimetry, nor did we perform hypothalamic thermoregulatory analyses, which limits our understanding of the neuroendocrine perturbations in the dynamics of BPAF-induced energy expenditure. Second, the study did not assess adipose tissue endocrine function, which is also important for energy intake as well as expenditure. Third, the lack of high-resolution lipidomics precludes a comprehensive description of tissue-specific lipid remodeling. Finally, interspecies differences in the toxicokinetics of BPAF may require more consideration when extrapolating conclusions. In conclusion, our findings demonstrated that BPAF exposure disrupted lipid metabolism homeostasis, leading to fat accumulation in adipose tissue and liver, especially under conditions of high-fat diet feeding. Metabolomics analysis further elucidated the relationship between altered lipid metabolism and changes in lipids levels induced by BPAF exposure. Protein phosphorylation analyses suggested that inhibition of the p-HSL signalling pathway might be responsible for the metabolic disturbances in adipose tissue. Collectively, this study demonstrated the potential obesogenic risk of BPAF via disrupting lipid metabolic homeostasis under both ND and HFD feeding conditions, which would provide scientific evidence for both the development of precision prevention strategy and promotion the safe use of BPAF.

## Data Availability

The original contributions presented in the study are included in the article/**Supplementary Material**. Further inquiries can be directed to the corresponding authors.
