# Supplementary figures and images for: Chronic BPAF exposure differentially enhances fat deposition in mice fed normal or high-fat diets via lipid metabolism dysregulation
Source: Front Endocrinol (Lausanne). 2025 May 15;16:1571076. doi: 10.3389/fendo.2025.1571076 (PMC12119261; doi:10.3389/fendo.2025.1571076)

Fig 3G

ND

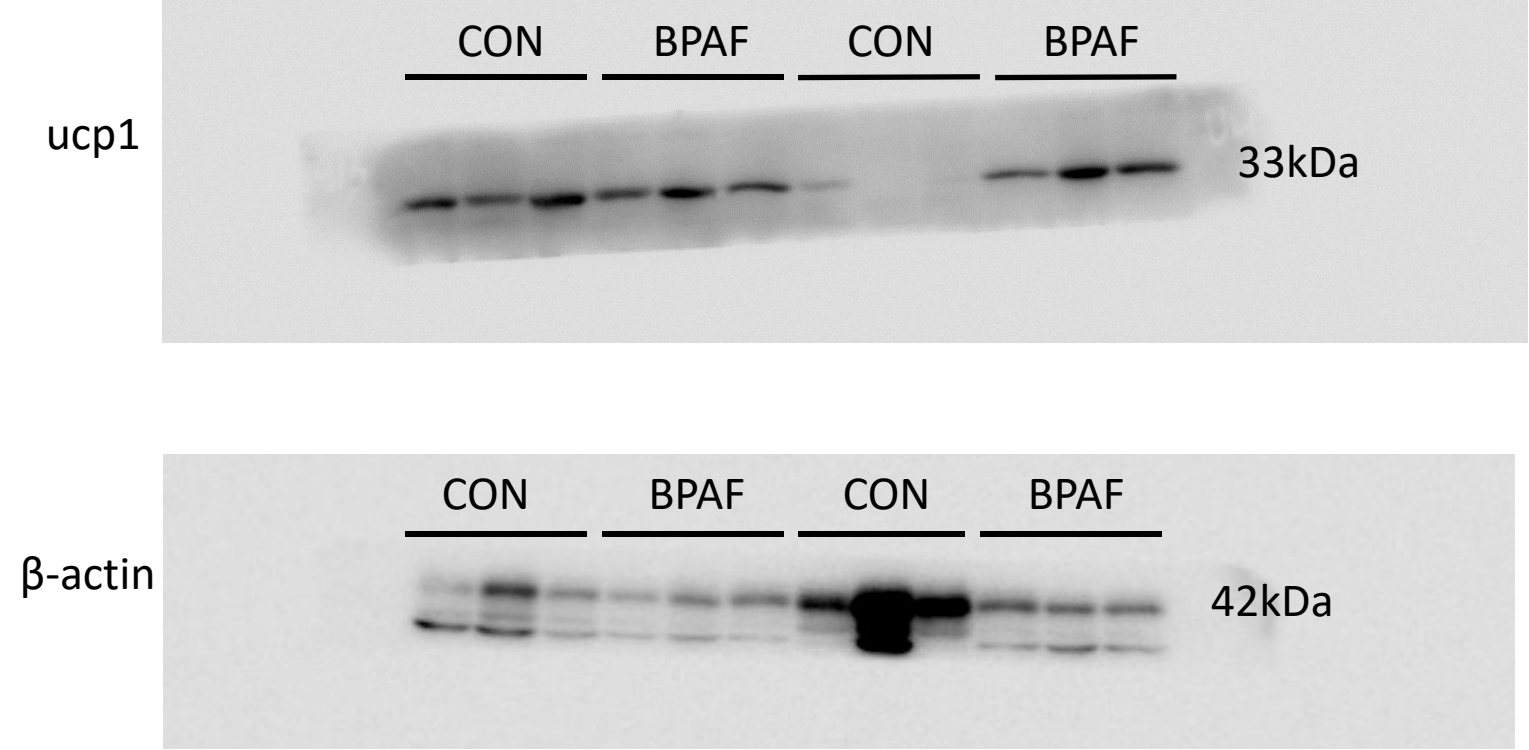

Fig 3I

HFD

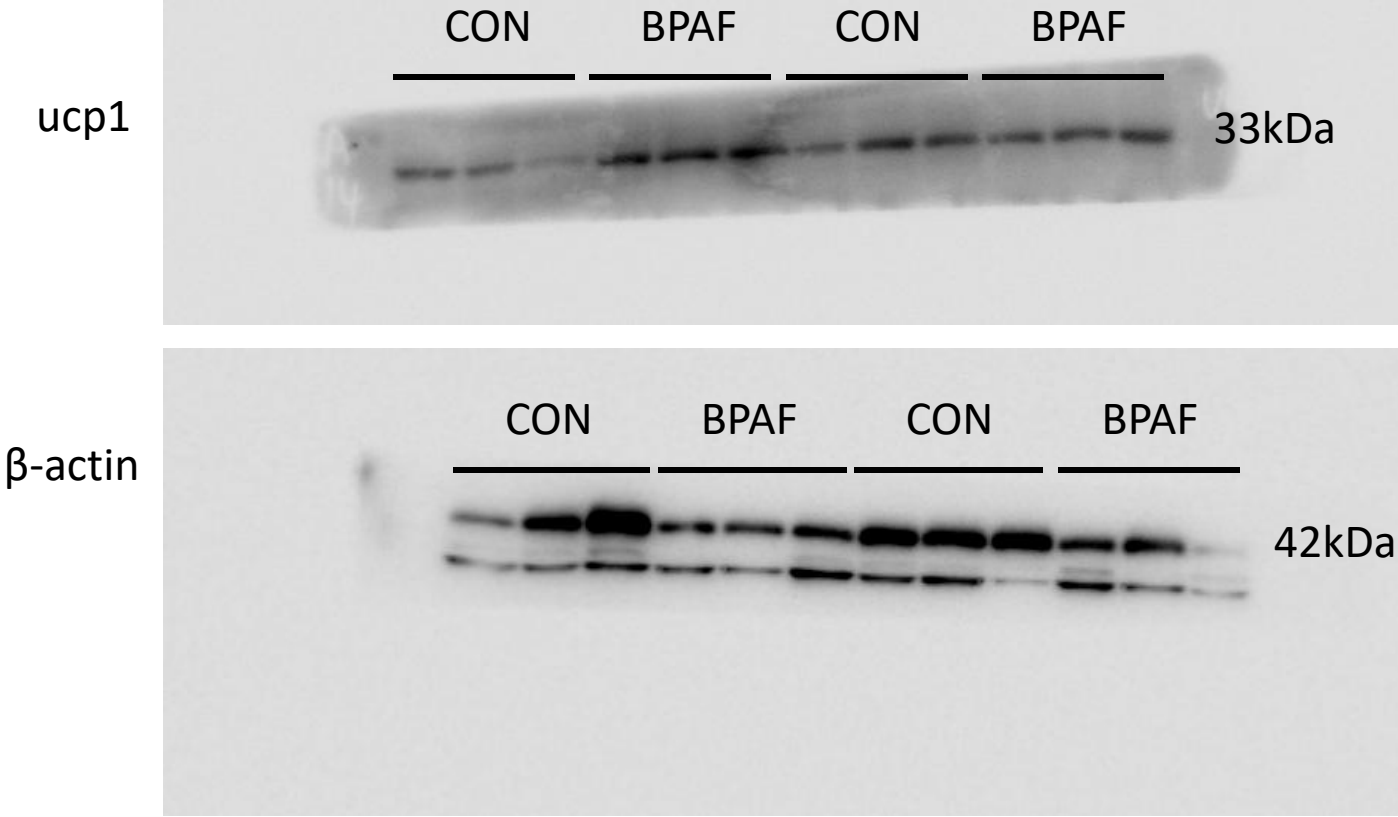

Fig 5E ND

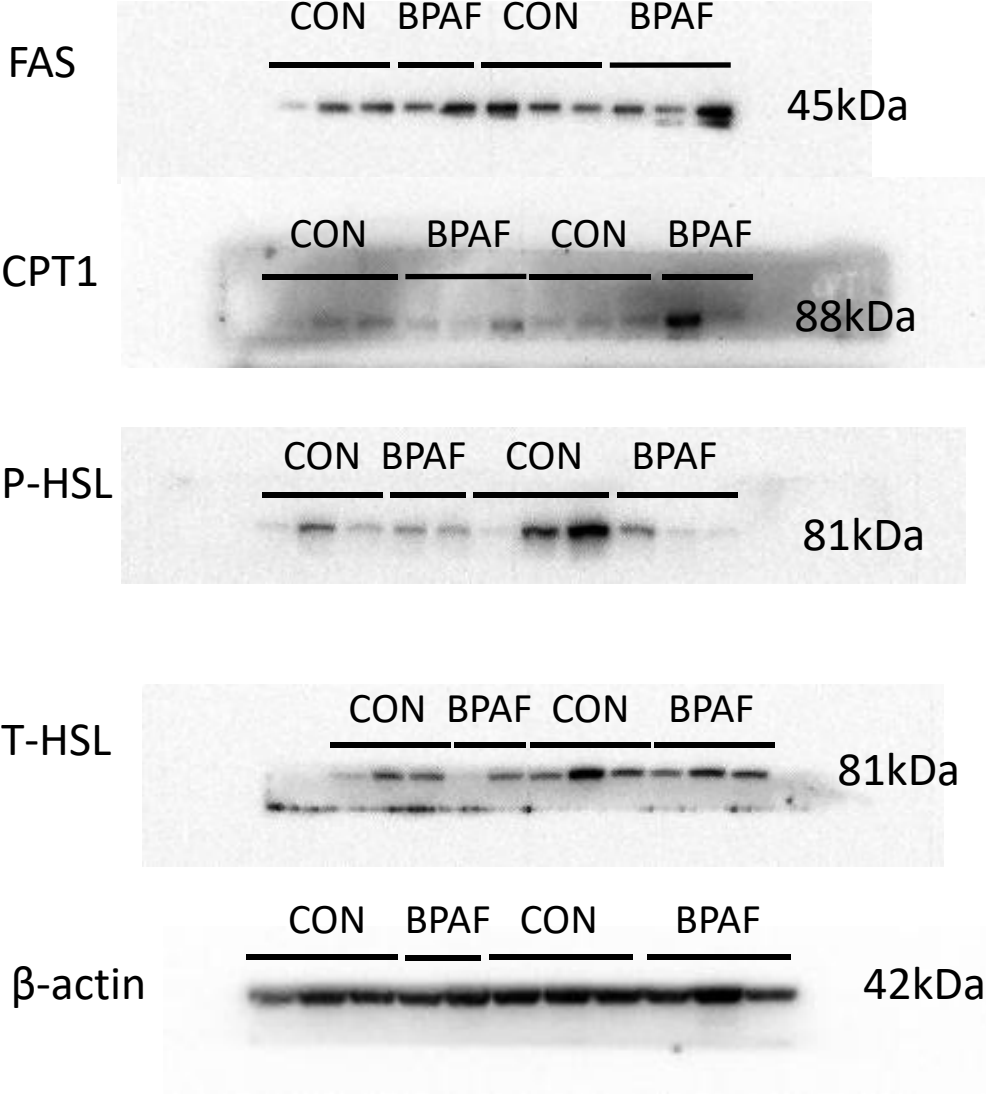

Fig 5F

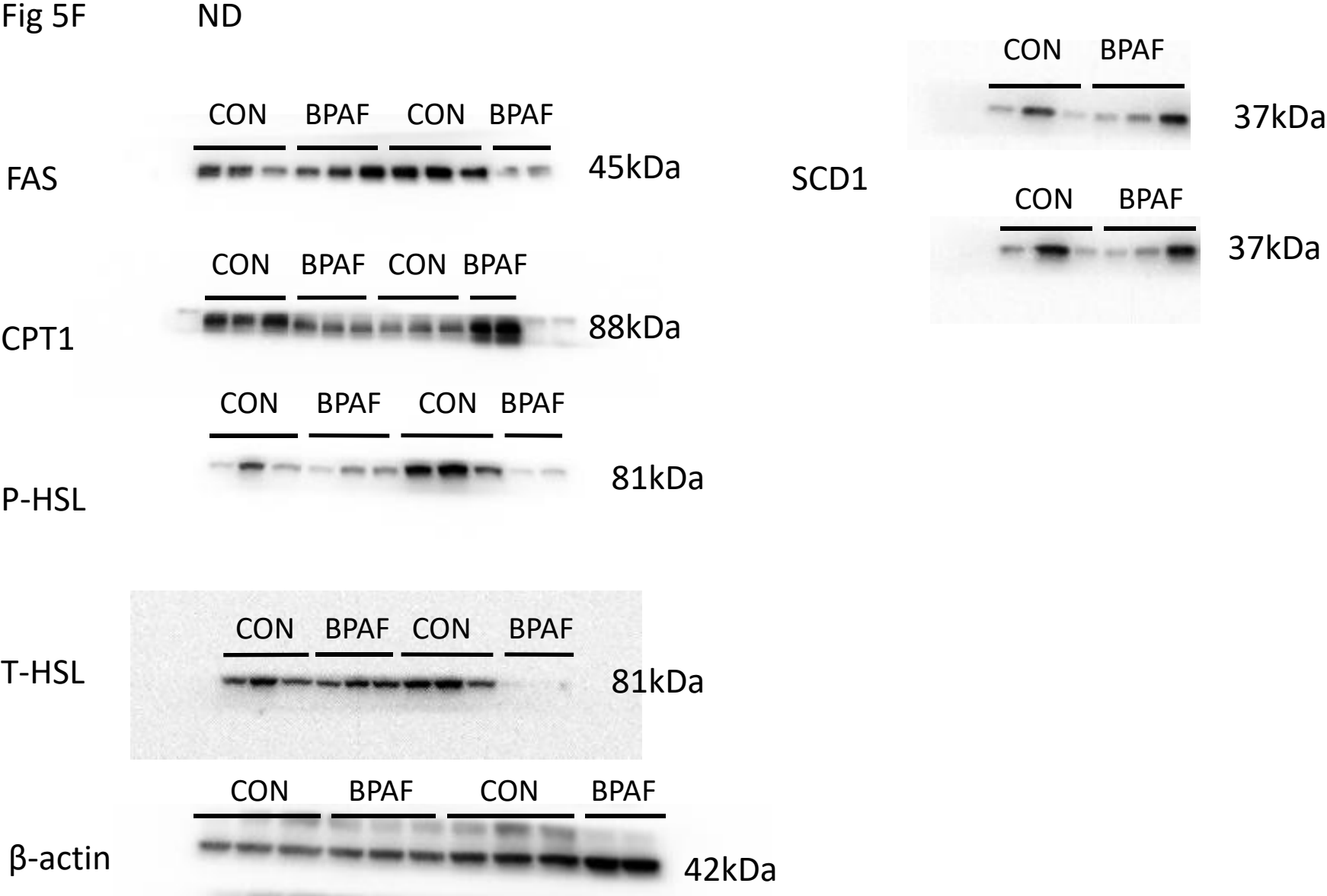

Fig 5G

HFD

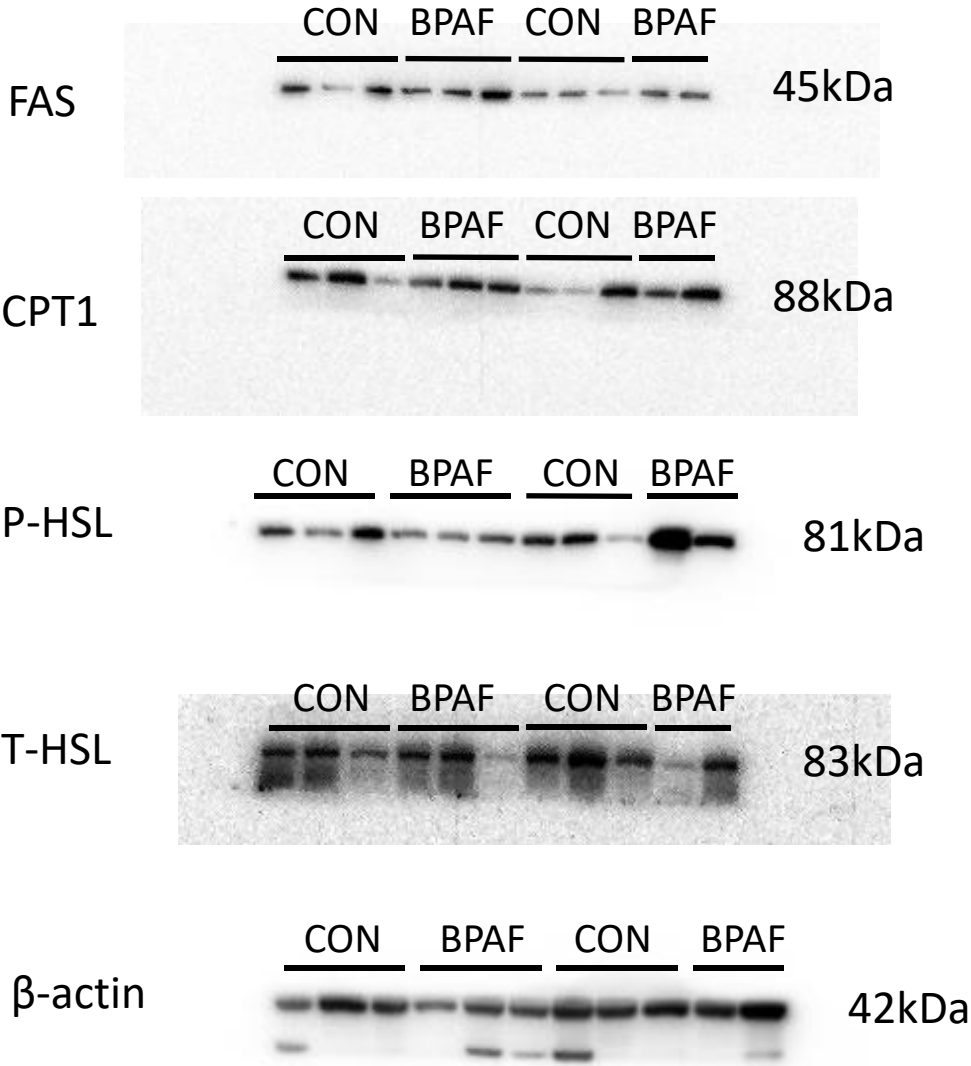

Fig 5H

HFD

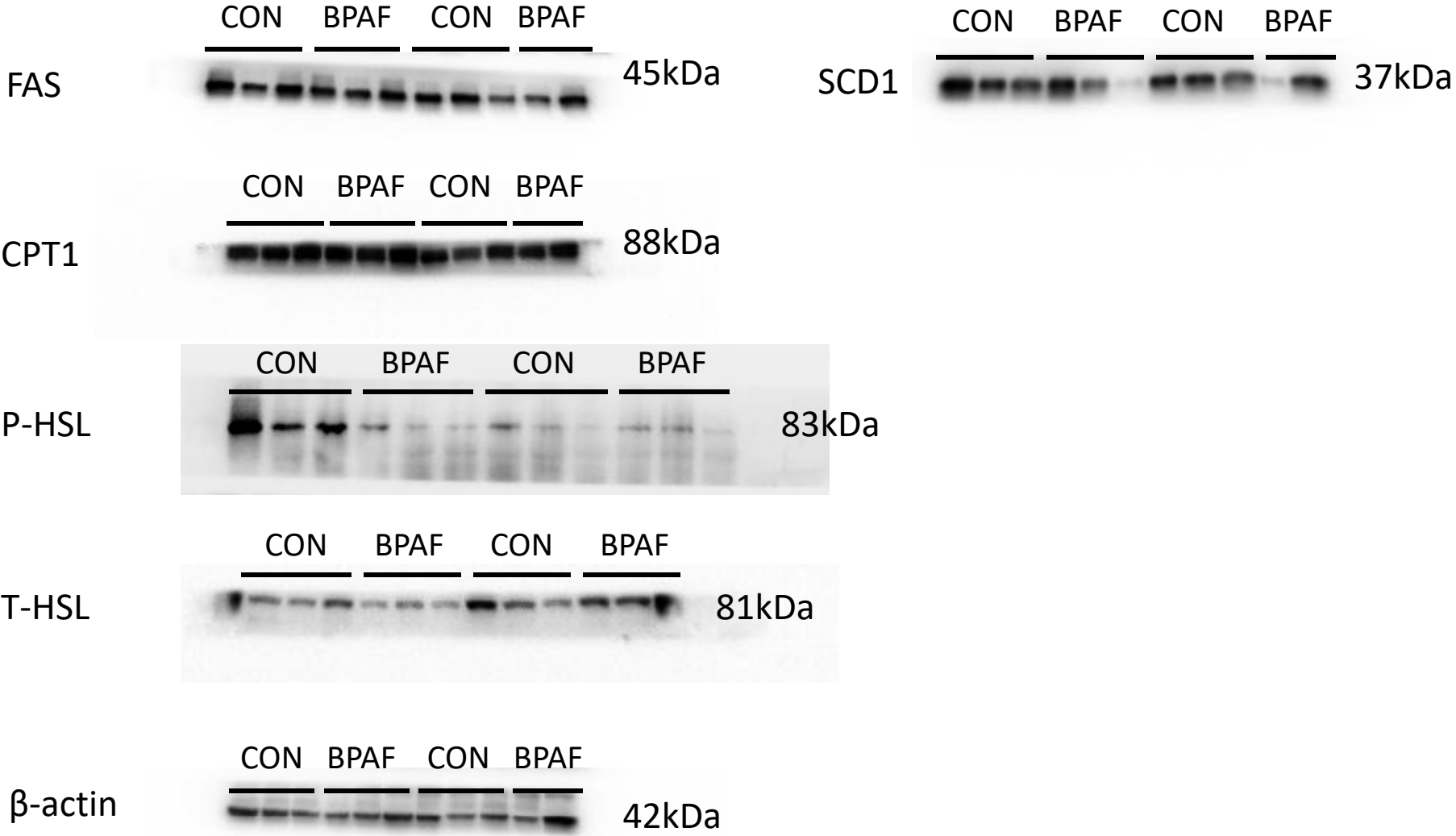

Supplement: Supplementary file 1 [file DataSheet1.pdf]
